#  Cone-beam tomographic analysis of canalis sinuosus accessory intraosseous canals in the maxilla

**DOI:** 10.3205/000261

**Published:** 2017-12-19

**Authors:** Abdalmalik O. Ghandourah, Ashkan Rashad, Max Heiland, Badr M. Hamzi, Reinhard E. Friedrich

**Affiliations:** 1 Department of Oral & Maxillofacial Surgery, University Medical Center Hamburg-Eppendorf, Hamburg, Germany; 2 Department of Oral & Maxillofacial Surgery, University Medical Center Charité, Berlin, Germany

**Keywords:** CBCT, canalis sinuosus, accessory canals, maxilla, implant

## Abstract

The aim of this study was to assess the frequency, location and width of accessory canals (AC) of canalis sinuosus (CS) using cone beam computed tomography and compare our findings with recent literature. Additionally, intraosseous canals (IOC) in the sinus wall other than the CS were noted. A retrospective analysis of 219 scans from our university department was conducted. The registered parameters were age, sex, location and width of canals. Group A consisted of 201 (85 males and 116 females) adults ranged from 19 to 99 years of age (mean age = 47.5 years). A total of 136 patients (67.6%) presented at least 1 AC, of which 55 cases showed a foramen width greater than 1 mm (27.4%). Group B had a sample size of 18 adolescents (7 males and 11 females) with a range of age from 7 to 18 years (mean age = 15.8 years). Eight cases (44.4%) presented at least one AC, of which only 3 had a foramen width greater than 1 mm (3.6%). ACs were found to occur predominantly at central incisors region (Group A) and the left lateral incisor and canine as well as the central incisors regions (Group B). Adolescents showed a lower prevalence of accessory canals compared to adults. These findings supplement earlier reports on the anatomical variations of the intraosseous vessel and nerve conduits of the maxilla. Surgical interventions in this area can be planned more precisely, taking into account the three-dimensional imaging, thus possibly protecting these sensitive structures.

## Zusammenfassung

Ziel dieser Untersuchung war die Einschätzung von Häufigkeit, Lage und Lumenweite akzessorischer Kanäle (AK) des Canalis sinuosus (CS) anhand dreidimensionaler Schnittbilder dieser anatomischen Struktur in der digitalen Volumentomographie (DVT). Die Ergebnisse sollten mit Daten der aktuellen Literatur verglichen werden. Zusätzlich wurden auch weitere intraossäre Kanäle der Kieferhöhlenwand registriert und vermessen, die sich vom CS abgrenzen ließen. Diese retrospektive Untersuchung wurde an 219 Schnittbildserien der Universitätsklinik durchgeführt. Es wurden Alter, Geschlecht, Lokalisation der Kanäle und deren Weite registriert. In Gruppe A wurden 201 Erwachsene (85 Männer und 116 Frauen) zusammengefasst (Alter: 19 bis 99 Jahre, Mittelwert (MW): 47,5 Jahre). Bei 136 Patienten (67,6%) lag zumindest ein akzessorischer Kanal vor. Von dieser Gruppe hatten 55 Patienten ein Foramen mit einem Durchmesser größer als 1 mm (27,4%). Gruppe B bestand aus 18 Heranwachsenden (7 Männer und 11 Frauen) mit einer Altersspanne von 7 bis 18 Jahre (MW: 15,8 Jahre).

Acht Fälle (44,4%) zeigten zumindest einen AK, von denen allerdings nur 3 ein Foramen mit einer Weite von über 1 mm aufwiesen (3,6%). AKs wurden vornehmlich in der Region der zentralen Incisivi nachgewiesen (Gruppe A), dagegen in Gruppe B auch im Bereich der seitlichen Incisivi und Canini wie auch in der Region der zentralen Incisivi. Die Gruppe der Heranwachsenden zeigten eine geringere Prävalenz der AKs im Vergleich zu der Gruppe der Erwachsenen. Die eigenen Befunde ergänzen frühere Berichte über die anatomische Variation der intraossären Gefäß- und Nerven-Kanäle des Oberkiefers. Chirurgische Eingriffe in diesem Bereich können unter Anwendung der DVT deutlich präziser geplant werden. Fallweise können mit dieser dreidimensionalen Bildgebung diese empfindlichen Strukturen während des Eingriffs geschont werden.

## Introduction

Knowledge of aberrant anatomy is necessary to avoid misdiagnosis [1]. In reported cases, a wide projection from the canalis sinuosus (CS) appeared as a radiolucent area in panoramic and periapical radiographs, which mimicked an inflammatory lesion [2], [3], [4]. In another case report, the author described “wide bilateral canals” that communicated with the palate at the canine-premolar region on a computerized tomography (CT) image. These canals were not visible on a panoramic radiograph [5]. In two clinical cases patients had received dental implants at the anterior maxilla near an accessory canal (AC). They suffered from postoperative pain. One of whom experienced immediate pain relief after explantation [6]. Hemorrhage and neurosensory disturbances are the most common complications related to implant surgery [7]. AC which carry neurovascular supply should be considered when planning for implant therapy as contact with nervous tissue may cause failure of osseointegration of dental implants [3], [8]. Awareness of the presence and location of ACs will help in performing complication-free procedures. The purpose of this study was to assess the frequency, location and width of ACs using cone-beam computed tomography (CBCT) and compare our findings with recent literature.

## Material and methods

### Study groups

A total of 219 patients were included and divided into two age groups. The study groups comprised adults (Group A) and adolescents (Group B). A retrospective analysis of midface cone-beam computed tomography (CBCT) scans, collected from the database of our university department of oral and maxillofacial surgery, were conducted to determine the frequency, location, and width of intraosseous canals (IOC). It concerned namely the CS which originates laterally or inferior from the infraorbital nerve and passes antero-infero-medially to the nasal pyriform aperture (NPA) [9] and its accessory branches at the alveolar process level. ACs were documented for location and width. The number and symmetry of other IOCs located in the sinus wall were also noted. Our inclusion criteria were the absence of malformations, bone lesions, dental implants in the anterior maxilla, and fractures. Structures suspected of being canals but measured less than 0.5 mm in diameter were not considered. 

### Radiographic analysis

Radiographs were taken through a CBCT scanner (3D Accuitomo 170, Modell MCT-1 EX-1 F17; Morita MFG Corp., Kyoto, Japan). Images were carried out in “standard mode” with an exposure time of 18 seconds (360°) and thickness of 1 mm slices, voxel size was 80–250 µm, and exposure volume 170 mm x 120 mm. Scans were analyzed using OsiriX MD™ as a viewing software. Investigations were carried out in a darkened room using a diagnostic monitor with a resolution of 2560 x 1440 p. All scans were descriptively analyzed and measured by the same observer twice with a period of 2 months between evaluations. The radiographic analysis was carried out as follows: First, the CS and other IOCs in the sinus wall were identified in the coronal view. The CS was measured at the NPA. Due to the variations in CS morphology, this area seemed the most reproducible. Afterward, the presence of AC was evaluated in the sagittal view. Finally, all measurements were conducted in the axial view at the closest distance of intraosseous canal diameter. Measurements were described as either being smaller or greater than 1 mm. The location of ACs was defined by dividing the area of interest into five regions as follows: central incisors, right lateral incisor and canine, left lateral incisor and canine, right premolars and left premolars.

### Statistics

The collected data of 201 adults and 18 adolescents was analyzed. The distribution of categorical data was described by absolute and relative frequencies. To compare the frequency distributions of two independent groups the Fisher’s exact test was carried out. Due to the explorative nature of this study, the p-values are interpreted descriptively. A p-value of <0.05 was considered as significant.

## Results

**Figure 1:**
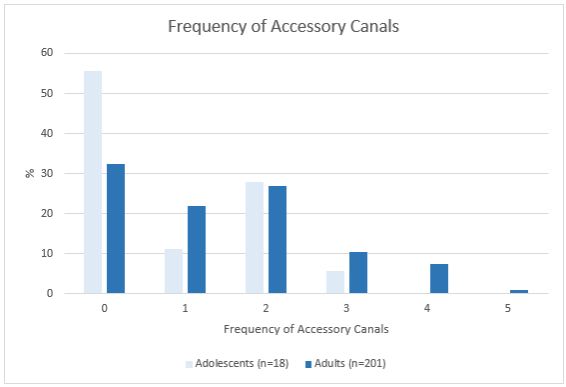
Illustration of the frequency of accessory canals for both studied groups. Total adolescents=18; total adult=201.

**Figure 2:**
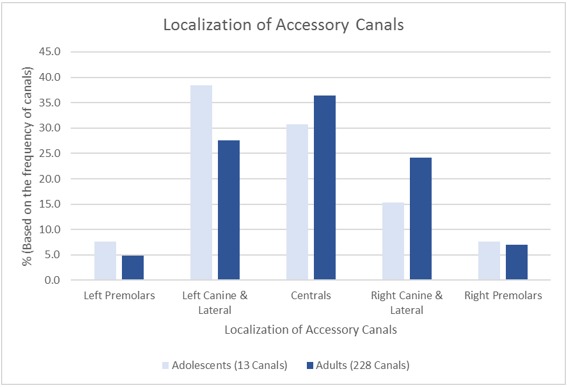
Localization of accessory canals in maxilla for both studied groups. Total canals in adolescent group = 13; total canals in adult group = 228.

The CS was registered on both sides in (100%) of the sample. From the total sample size 49 scans, (22.3%) had at least one additional IOC in the sinus wall other than the CS. A total of 27 CBCTs showed asymmetry of IOCs in the sinus wall, 13 CBCTs exhibited asymmetry in course while having the same number of canals on both sides, whereas the other 14 CBCTs showed an uneven distribution of the number of canals on either side. Although ACs were detected in less than half of the adolescent group (44.4%), it occurred in approximately two-thirds of the adult group (67.7%) and were found to appear in the region of the anterior teeth more frequently in both groups. There was no significant relation between sex and presence. However, age showed a clear difference in the prevalence of ACs. See Table 1 (Tab. 1), Table 2 (Tab. 2), Figure 1 (Fig. 1), Figure 2 (Fig. 2).

### Adult data

The data of 201 adults was described (85 males and 116 females) CBCTs. The age group ranged from 19 to 99 years of age (mean age = 47.5 years). In 136 cases, at least 1 AC (67.6%) was present, and a total of 285 ACs were documented. In 55 patients, a foramen width greater than 1 mm (27.4%) was found (95% confidence interval [21.3%; 34.1%]). 

The locations of ACs were not equally distributed. The most common site was "centrals", and least likely at right and left bicuspids. In (82.1%) the CS was measured to be ≥1 mm. The same number of left and right IOC in the sinus wall was documented for 188 adults (93.5%, 95% confidence interval [89.2%; 96.5%]) see Table 3 (Tab. 3) and Table 4 (Tab. 4). Out of 188 adults 13 adults (6.9% of 188) showed an asymmetrical course, and 175 showed a symmetrical course of the canals (93.1% of 188, 95% confidence interval [88.5%; 96.3%]).

### Adolescents data

**Figure 3:**
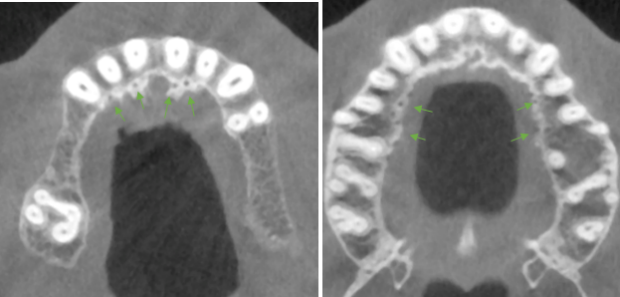
Example of accessory foramina in the axial view

**Figure 4:**
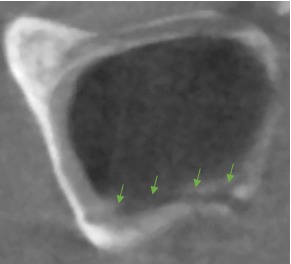
A sagittal view of the sinus showed an IOC in the lateral sinus wall

**Figure 5:**
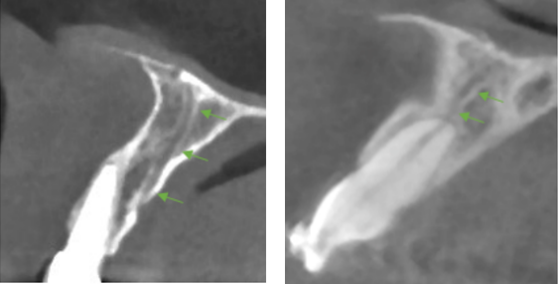
Examples of an accessory canal in the sagittal view

**Figure 6:**
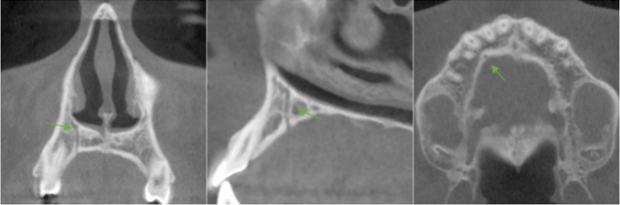
Showing a well-defined accessory canal in all three views

**Figure 7:**
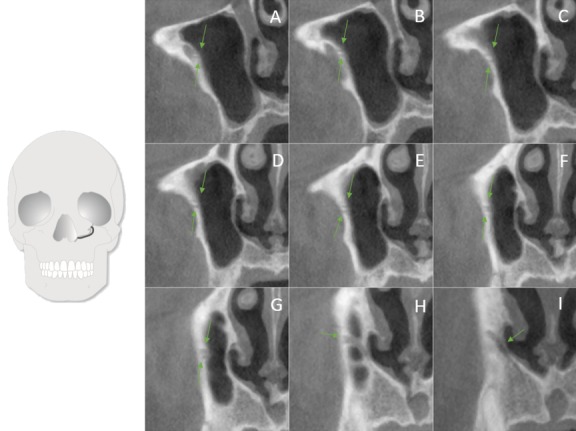
Two branches of the ASAN running parallel to each other and joining before reaching the NPA (from posterior to anterior)

**Figure 8:**
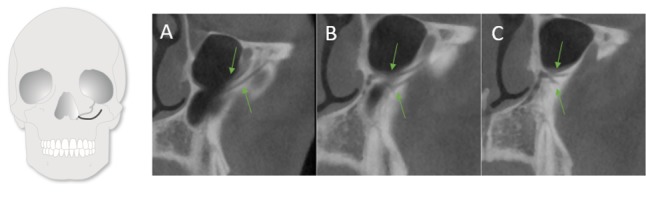
IOC originated lateral to the CS and joined it at the NPA (from posterior to anterior)

**Figure 9:**
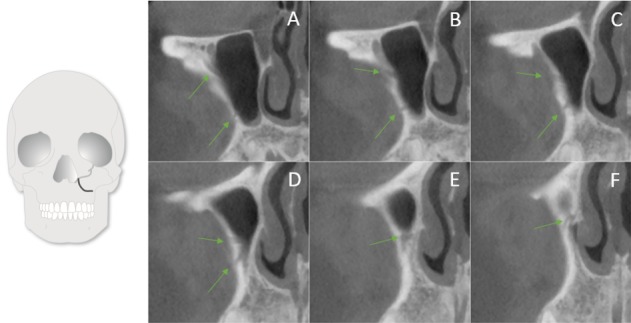
A buccal IOC joining the CS at NPA (from posterior to anterior)

The data of 18 adolescents was described (7 males and 11 females). The age group ranged from 7 to 18 years (mean age=15.8 years). Eight cases presented at least 1 AC (44%) and a total of 15 ACs were identified, with only 3 cases showing an AC with a foramen width greater than 1mm (16.7%, 95% confidence interval [3.6%; 41.4%]) see Table 5 (Tab. 5) and Table 6 (Tab. 6). The locations are not equally distributed. The most common sites are "left canine and lateral incisor" and "central incisors". Least likely sites are the right and left bicuspids. In (77.8%) the CS was measured to be ≥1 mm. The same number of left and right IOC in the sinus wall was documented for 17 adolescents (94.4%, 95% confidence interval [72.7%; 99.9%]). All 17 showed a symmetrical course of the canals (100% of 17, 95% confidence interval [80.5%; 100%]). The radiological findings are illustrated in Figure 3 (Fig. 3), Figure 4 (Fig. 4), Figure 5 (Fig. 5), Figure 6 (Fig. 6), Figure 7 (Fig. 7), Figure 8 (Fig. 8), Figure 9 (Fig. 9).

## Discussion

The present study of 201 CBCT scans of group A registered at least one AC in 136 cases (67.6%). This result is higher than results reported by previous studies of 51.7%, [6], 55.1% [10] and 15.7% [11]. These discrepancies could be attributed to differences in voxel size. Additionally, de Oliveira-Santos et al. [11] only reported canals with a diameter of ≥1 mm. Furthermore, when comparing the prevalence of ACs with a diameter of ≥1 mm, the results of the current study (27.4%) coincide with those reported by von Arx et al. (27.8%) [10]. There was no statistical significant difference observed between occurrence of AC and gender. This finding is in line with published results except Machado et al. [6] who found an increased prevalence of males over females, and is in contrast to the result reported by Sekerci et al. [12] who reported a higher incidence in females over males. 

Group B registered at least one AC in 44%, of which only 16.7% exhibited a diameter of ≥1 mm. There is a slight difference to the result reported by Sekerci et al. [12] of (22.3%) when comparing AC with a diameter of ≥1 mm; this could be credited to the difference in the sample age, ethnicity and imaging parameters. Machado et al. [6] reported a (33.3%) occurrence rate in patients ≤20 years of age which is less than the results reported in this study but again could be related to voxel size differences. Regarding the relation between age and presence, this study confirms the results of Sekerci et al. [12] which reported a steady increase in occurrence with older patients. 

In the current study, the location of ACs was found in group A to occur predominantly in the region of the central incisors (41.3%). These results are similar to the report given by von Arx et al. [10] (56.7%). In decreasing frequency follow the left and right lateral incisors and canine regions (31.3% and 27.4%, respectively) and the finding is rarely seen in the left and right premolar regions (5.5% and 4.6%, respectively). The location differed slightly in group B. The area of high occurrence was the left lateral incisor and canine region (27.8%). This finding is similar to the report by Sekerci et al. [12]. However, the second highest region of occurrence in this study was the central incisors region (22.2%) which does not correlate to the mentioned study. Probably caused by observer subjectivness as the canals are often located palataly and could be difficult to attribute to the lateral or central incisors. The ACs from the CS were reported to have a mean diameter of 1.31 mm [10]. 

The CS was described as transmitting the anterior superior alveolar nerve (ASAN) and vessels [13]. Von Arx and Lozanoff [9] studied the ASAN and reported that it originated at a mean distance of 12.2 ± 5.79 mm posterior to the infraorbital foramen. And it was described to be between one-half and one-third that of the infraorbital nerve, it courses laterally to the orbital margin and then passes antero-infero-medially below the infraorbital foramen to the NPA. The ASAN was 2.8 ± 5.13 mm lateral to the infraorbital foramen and ran infero-antero-medially to the NPA at a mean distance of 5.5 ± 3.07 mm inferior to the infraorbital foramen. On average, the ASAN was 13.6 ± 3.07 mm superior to the nasal floor and had a mean distance of 4.3 ± 2.74 mm laterally to the nasal aperture, and 3.3±2.60 mm at the nasal floor. All ten dissection cases showed the ASAN encased in a thin layer of bone, while Robinson and Wormald reported dehiscence in 12.5% of 40 maxillae dissections [14]. Moreover, evidence of direct anastomosis between the ASAN and the greater palatine nerve has been described [15]. This study reports observing IOCs in the sinus wall other than the CS in 22.3% of the sample size. These IOCs are suspected of being the middle superior alveolar nerve (MSAN) and posterior superior alveolar nerve (PSAN) and artery. 

The MSAN originated from the posterior one-third of the infraorbital nerve at a mean distance of 2–8 mm from the posterior end of the infraorbital canal/groove. It traveled in the lateral sinus wall antero-inferiorly behind the root of the zygoma to reach the superior alveolar nerve plexus in the premolar region [16], [17]. Variability was also observed and categorized into five types. Type 1 was an instance of premature origin from the maxillary nerve, while types 2–4 originated from the infraorbital nerve and ran respectively in the posterior, lateral and anterior walls of the sinus. Type 5 was described as “delayed separation” as it accompanied the ASAN and separated at the mid sinus, then coursed infero-laterally to the premolar region [17]. The middle superior alveolar nerve was found in 54% of cases with manifest variations occurring in 28% and absence in 18% [17] while Robinson and Wormald [14] reported the MSAN to be present in only 23% of their dissections.

The PSAN was found in all dissections by Heasman [16]. In 68% of autopsies, the posterior superior alveolar nerve gave two branches one of which accompanied the posterior superior alveolar artery, while the second fed the maxilla. The PSAN originated from the maxillary nerve after it traversed the foramen rotundum before it entered the infraorbital canal/groove. Then the PSAN coursed inferiorly innervating the gingiva and mucosa of the cheek. Finally, it entered its foramina at 10–43 mm superior to the most inferior point of the maxillary tuberosity. In 62.2% the PSAN traveled through the posterior alveolar canal in the lateral wall of the sinus. In 37.8% it coursed under the Schneiderian membrane and gave fine branches to innervate the membrane and molar teeth [18]. Maridati et al. [19] proposed the utilization of a double windows osteotomy technique for sinus lifts in cases where the alveolar antral artery was >2 mm and exhibited an intraosseous course. Findings of the reviewed literature are summarized in Table 7 (Tab. 7) and Table 8 (Tab. 8).

In conclusion, the current study confirms previous findings of the presence of ACs in the alveolar process which can be viewed by utilizing CBCT. ACs are not equally distributed with the most common area of occurrence being the central incisors region followed by the lateral and canines and lastly the premolar regions. There was no significant relation between sex and presence. However, age showed a clear difference in the prevalence of ACs. IOC in the sinus wall should be handled on a case by case scenario to offer the best treatment for the individual.

## Acknowledgments

Abdalmalik O. Ghandourah and Ashkan Rashad equally contributed to the article as joint first authors.

## Competing interests

The authors declare that they have no competing interests.

## Figures and Tables

**Table 1:**
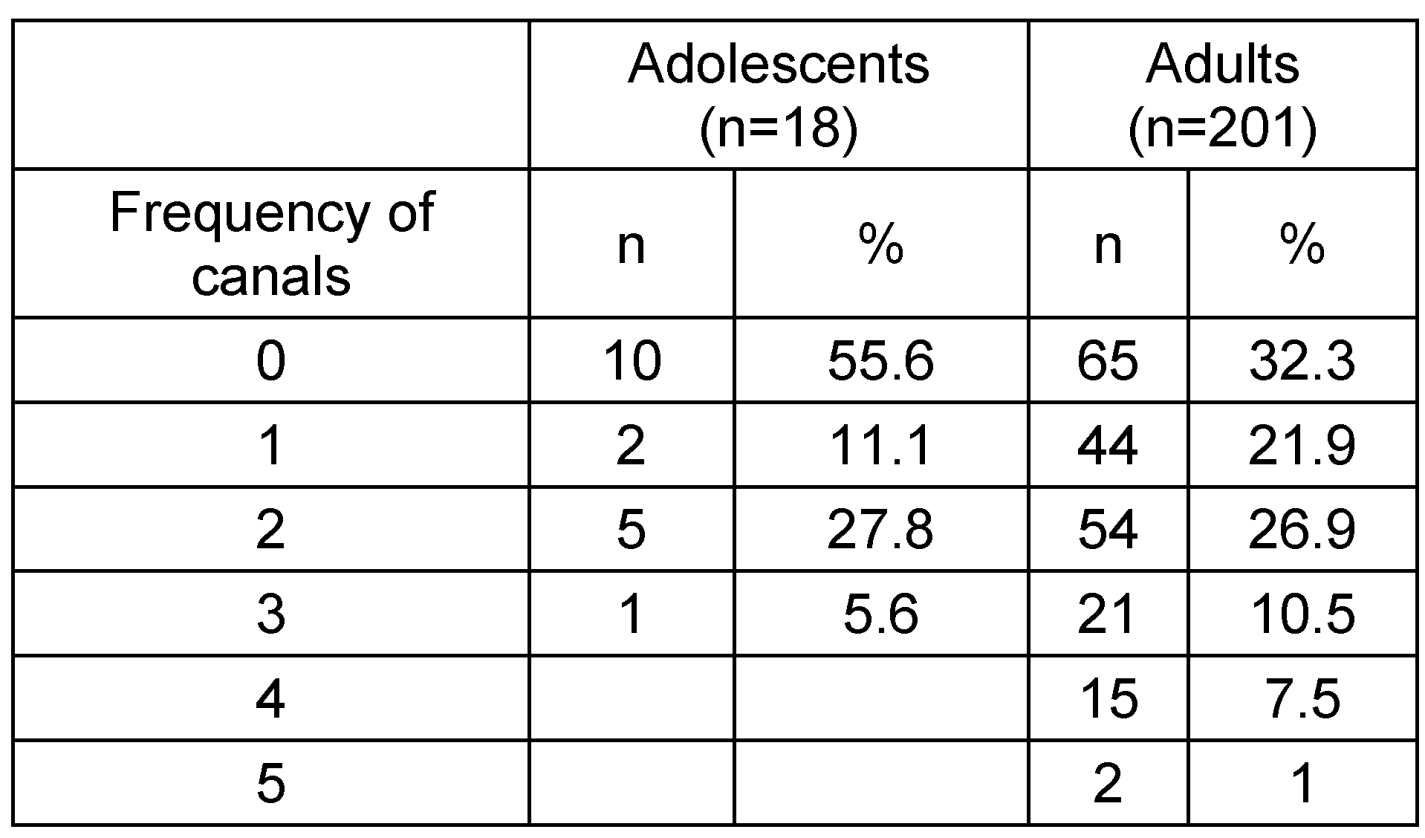
Frequency of accessory canals for both studied groups

**Table 2:**
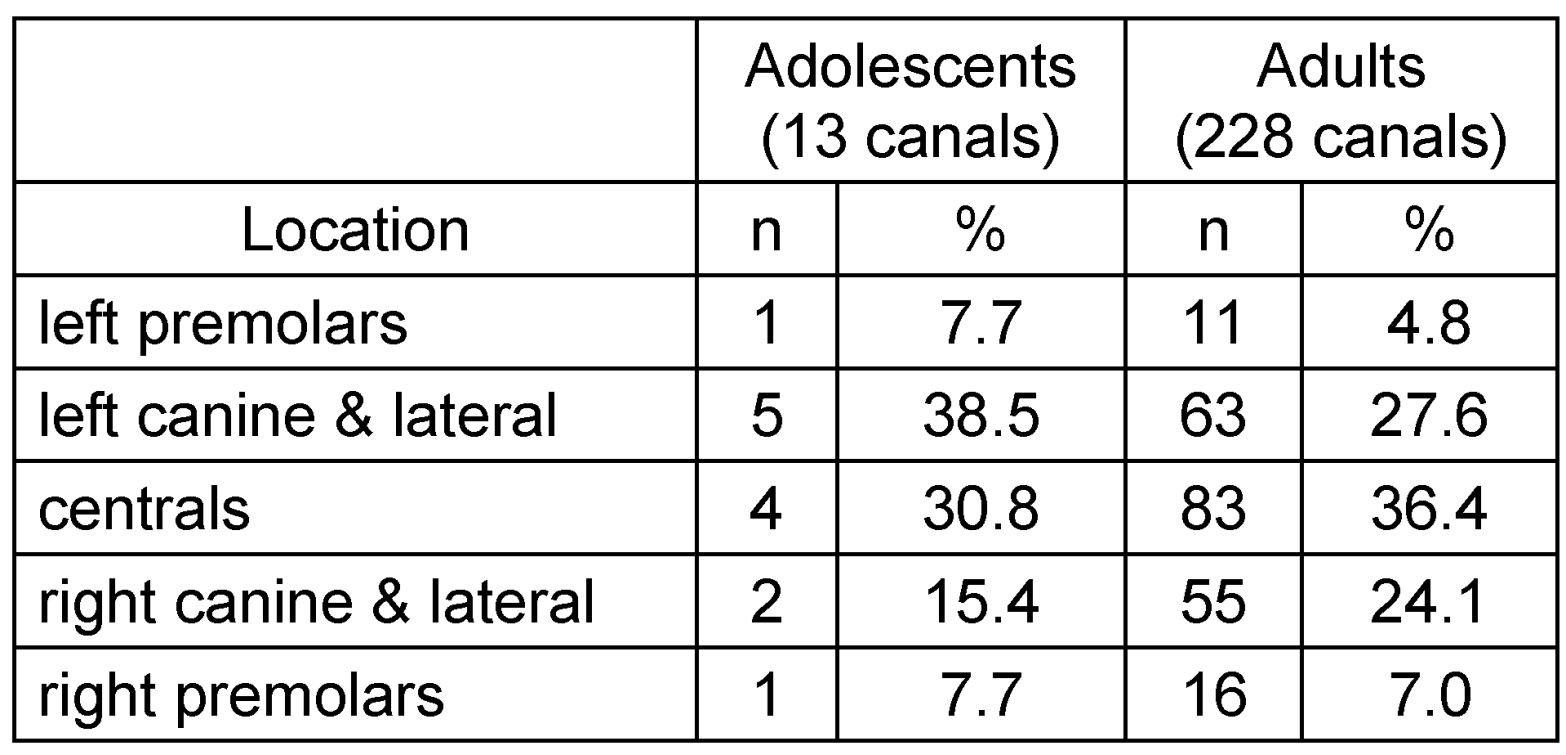
Localization of accessory canals in maxilla for both studied groups

**Table 3:**
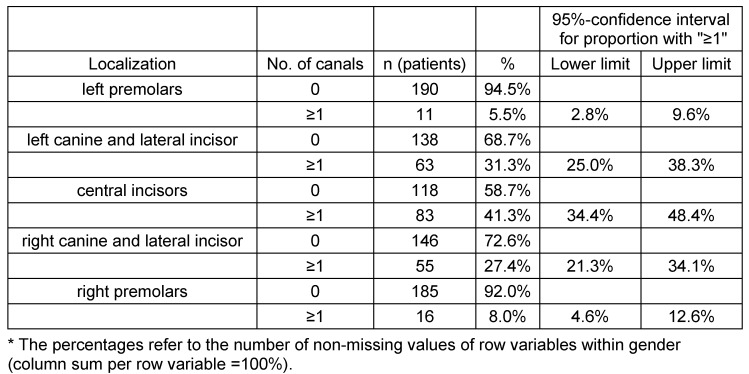
Absolute and relative frequencies of the localization of accessory canals with a 95% confidence intervals for the percentage of adults with at least one canal

**Table 4:**
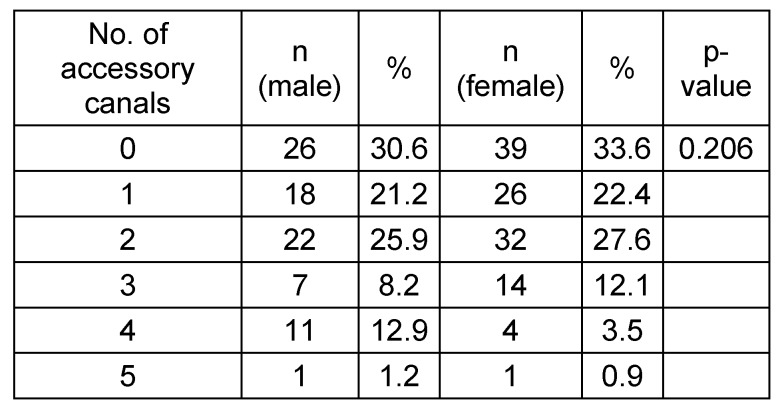
Absolute and relative frequencies of the number of accessory canals in adults depending on the gender. Total males n=85; total females n=116.

**Table 5:**
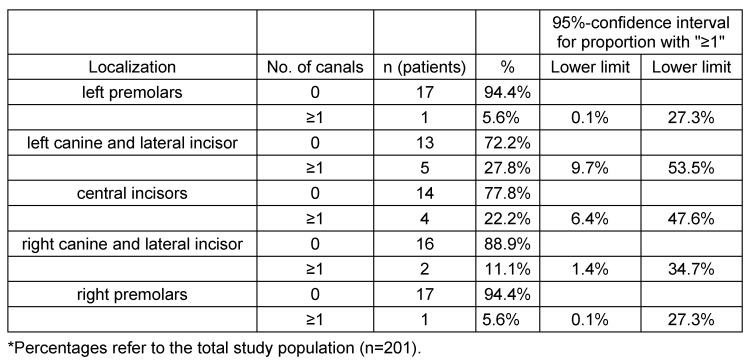
Absolute and relative frequencies of the localization of accessory canals with a 95% confidence intervals for the percentage of adolescents with at least one canal

**Table 6:**
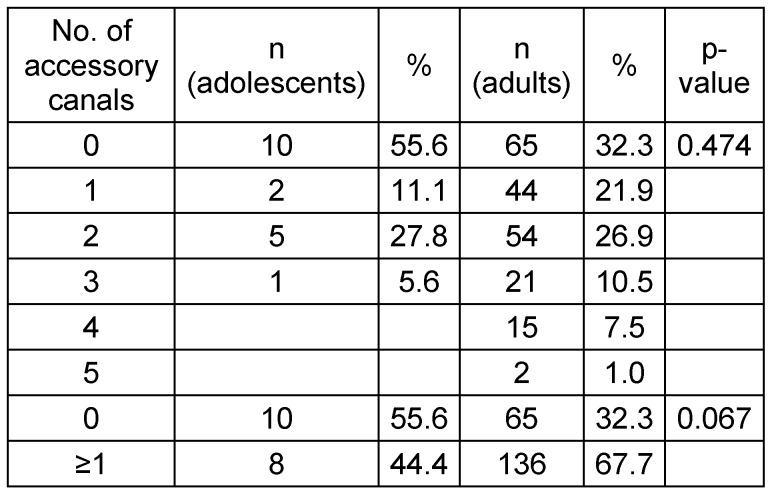
Absolute and relative frequencies of accessory canals depending on age groups. Total adolescents n=18; total adults n=201.

**Table 7:**
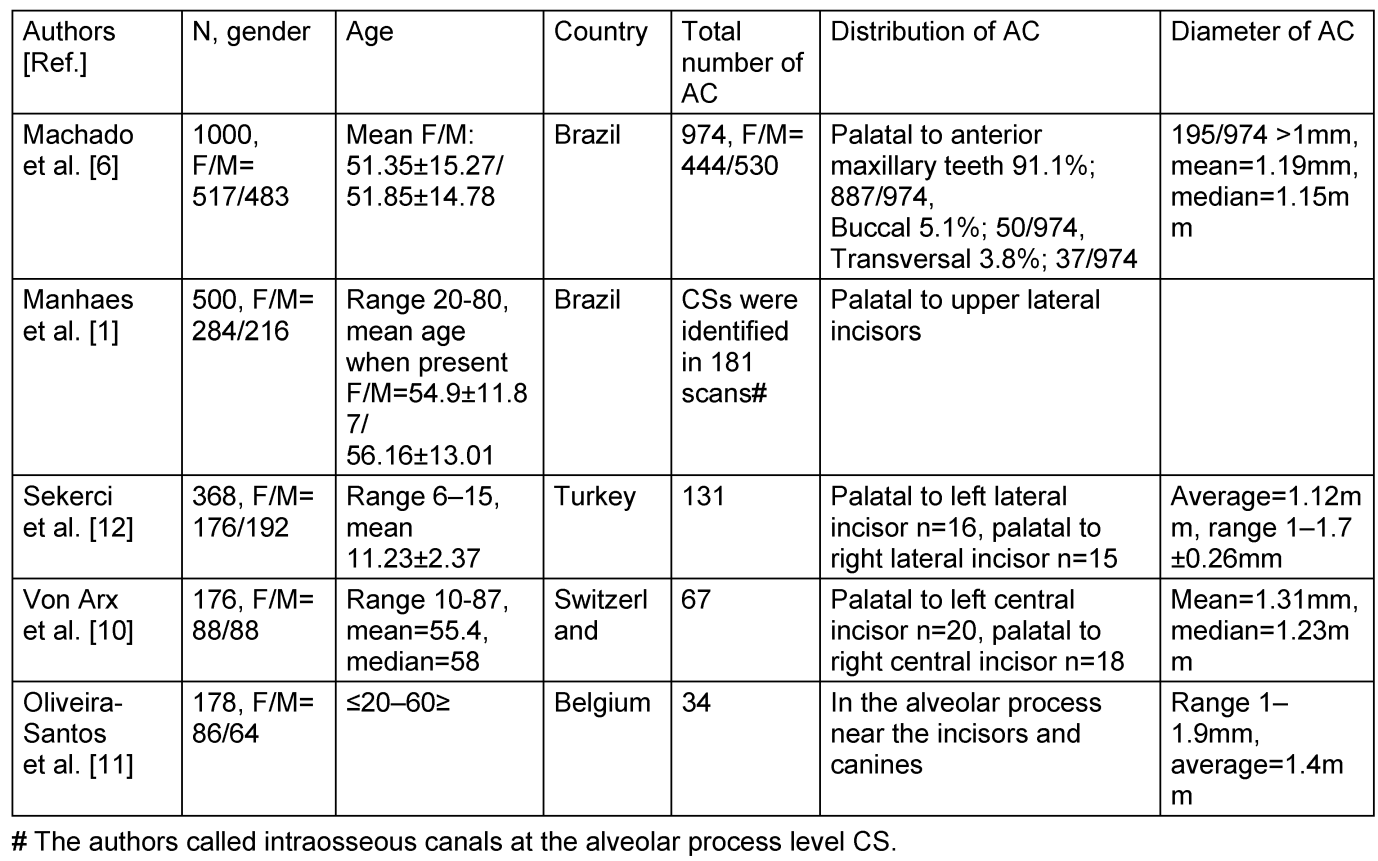
Literature review of radiological findings of accessory canals (AC) based on cone beam computed tomographic studies.

**Table 8:**
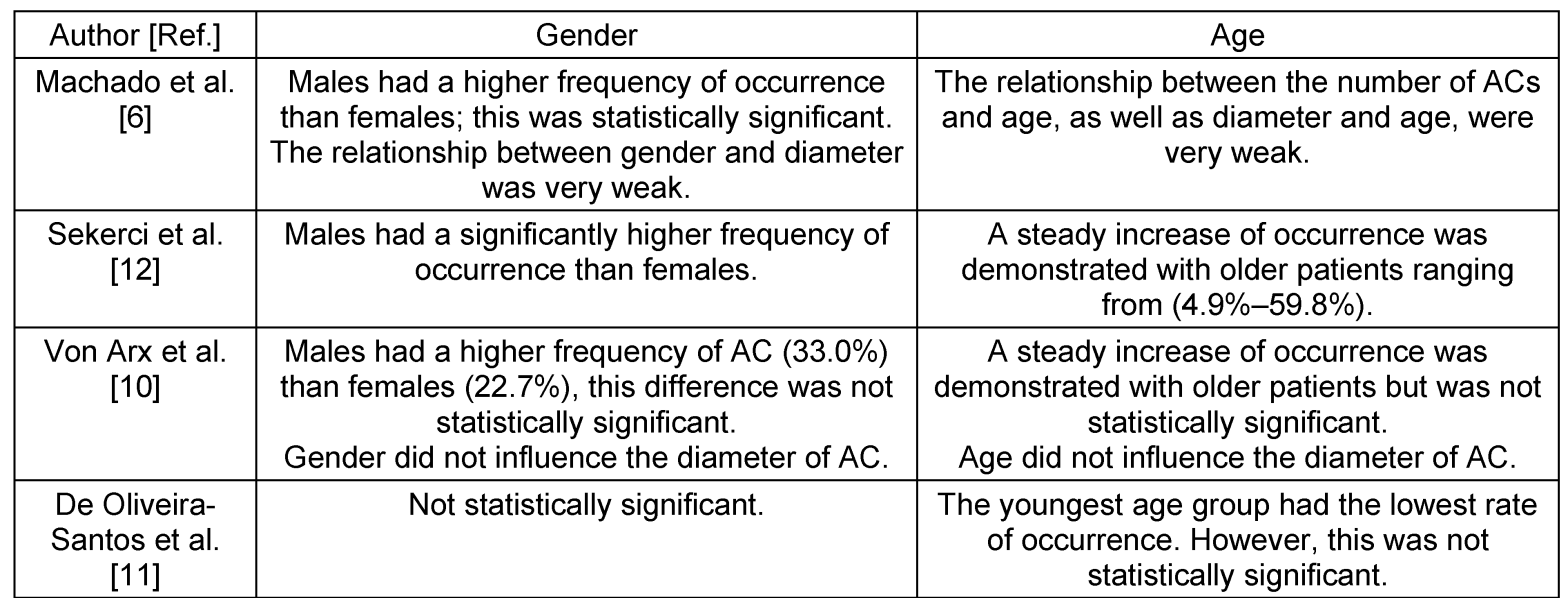
Literature review of the statistical significance of gender and age over the prevalence of ACs.
